# Identification and Characterization of Neoantigens As Well As Respective Immune Responses in Cancer Patients

**DOI:** 10.3389/fimmu.2017.01702

**Published:** 2017-11-30

**Authors:** Eva Bräunlein, Angela M. Krackhardt

**Affiliations:** ^1^Medizinische Klinik III, Klinikum rechts der Isar, Technische Universität München, Munich, Germany; ^2^German Cancer Consortium of Translational Cancer Research (DKTK), German Cancer Research Center (DKFZ), Heidelberg, Germany

**Keywords:** neoantigens, immunopeptidomics, T-cell responses, immune monitoring, adoptive T-cell transfer

## Abstract

Cancer immunotherapy has recently emerged as a powerful tool for the treatment of diverse advanced malignancies. In particular, therapeutic application of immune checkpoint modulators, such as anti-CTLA4 or anti-PD-1/PD-L1 antibodies, have shown efficacy in a broad range of malignant diseases. Although pharmacodynamics of these immune modulators are complex, recent studies strongly support the notion that altered peptide ligands presented on tumor cells representing neoantigens may play an essential role in tumor rejection by T cells activated by anti-CTLA4 and anti-PD-1 antibodies. Neoantigens may have diverse sources as viral and mutated proteins. Moreover, posttranslational modifications and altered antigen processing may also contribute to the neoantigenic peptide ligand landscape. Different approaches of target identification are currently applied in combination with subsequent characterization of autologous and non-self T-cell responses against such neoantigens. Additional efforts are required to elucidate key characteristics and interdependences of neoantigens, immunodominance, respective T-cell responses, and the tumor microenvironment in order to define decisive determinants involved in effective T-cell-mediated tumor rejection. This review focuses on our current knowledge of identification and characterization of such neoantigens as well as respective T-cell responses. It closes with challenges to be addressed in future relevant for further improvement of immunotherapeutic strategies in malignant diseases.

## Neoantigens as Highly Relevant and Attractive Targets of Tumor-Specific Immune Responses

Tumor immunologists have been fascinated on the possibility of tumor rejection by the immune system and recognition of tumors as “foreign” in comparison to healthy tissues for a long time. Tumor-associated antigens representing a group of antigens with accentuated but not unique prevalence in the tumor have been investigated as target antigens in a broad variety of tumor entities (1). However, therapeutic efficacy of such targeting approaches could be only rarely demonstrated (2) or has been accomplished outside of the self-educated T-cell receptor (TCR) repertoire (3). Central tolerance to self-antigens may represent one of the main reasons for the limited efficacy of such approaches. In contrast, tumor-specific antigens (TSA) are characterized by their unique presentation in tumor cells and, therefore, lack of negative thymic depletion of respective specific T-cell populations. Virus-associated antigens have traditionally been acknowledged as TSA in tumors with viral etiology as Merkel cell carcinoma, adult T-cell leukemia, and human papilloma virus (HPV)-associated tumors (4–6). In fact, HPV-induced tumors can be prevented by vaccinations and induced adaptive B-cell responses can be followed over years (7, 8). Mutations have been also early acknowledged to be highly interesting and potentially recognized by specific T cells (9–12), although the significance for a broader patient population remained elusive. A potentially more general role of mutations in tumor rejection has been demonstrated for a larger cohort of cancer patients only after introduction of immune checkpoint modulating antibodies, such as anti-CTLA4 and anti-PD-1, and association of the burden of non-synonymous mutations with response (13–16). Since then, neoantigens have become a major focus of interest either as potential biomarkers or as targets for directed immunotherapies. In fact, novel immunotherapeutic approaches targeting neoantigens by defined vaccines or directed T-cell transfer hold great promise to further improve therapeutic efficacy of immunotherapeutic approaches (17–21).

## Landscape of Non-Pathogen-Derived Neoantigens

**Figure 1:**
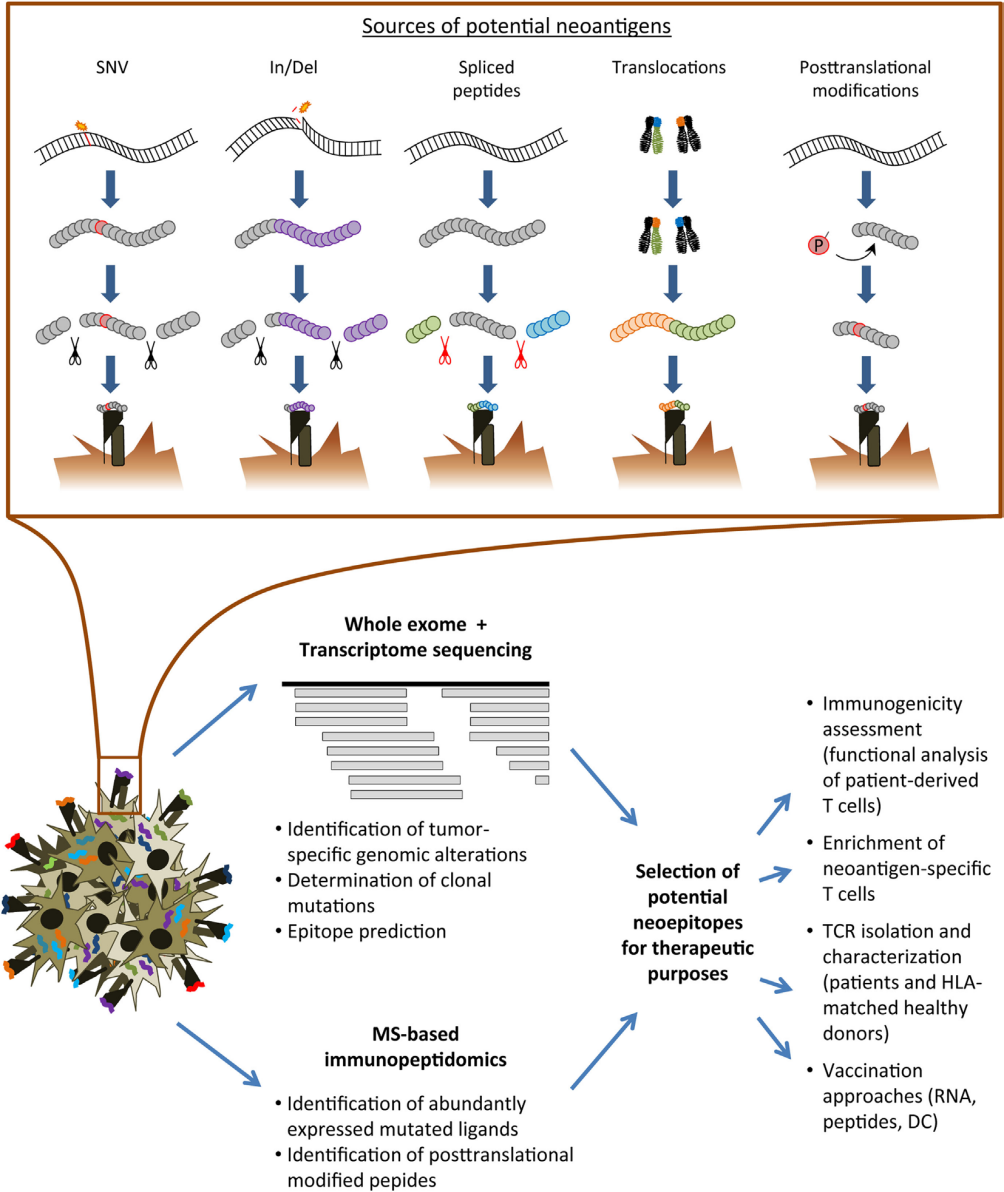
Overview of the neoantigen landscape and identification strategies. Upper row: sources of conceivable neoantigens exemplarily shown for HLA class I ligands. Lower row: schematic overview of analysis pipelines for the immunogenicity assessment of tumor-specific alterations. SNV, single nucleotide variant; In/Del, insertion/deletion; MS, mass spectrometry; TCR, T-cell receptor; HLA, human leukocyte antigen; DC, dendritic cell.

Currently, a diversity of tumor-specific alterations may serve as suitable sources for non-pathogen-derived neoantigens (Figure 1). Single nucleotide variants (SNV) resulting in non-synonymous substitutions have been a major focus of interest since a correlation of the non-synonymous mutation burden within the tumor and response to checkpoint modulators has been established (13, 14, 22). SNVs are typically present in malignancies induced by ultraviolet light exposition or tobacco smoke (23–25). Most of the SNV-derived neoantigens gain their immunogenic foreignness throughout altered amino acids involved in direct T-cell contact although also anchor positions may be affected resulting in potential lack of presentation of the wild-type peptide (26). Recurrent mutations may serve as public neoantigens enabling the development of targeted approaches applicable to broader patient cohorts (27–29). Nonetheless, the majority of immunogenic mutations appear to derive from patient-specific alterations. In addition to the potentially singular nature of a mutated peptide ligand, immunogenic neoantigens derived from non-synonymous mutations have been reported to be enriched for a distinct tetrapeptide signature homologous to epitopes derived from pathogens as suggested by data from Snyder and colleagues (13). However, subsequent studies could not confirm a prevalent role of such a defined peptide motif (22, 30).

Frame shifts in antigen-coding regions due to insertions or deletions have been described as additional promising source of TSA (31, 32). A recent report indicated frameshift-derived mutations to be enriched especially in cancer entities known to respond to immune checkpoint modulators and predicted neoantigens derived from these mutations correlated with response to immune checkpoint modulation as well as upregulation of immune signatures (33). Due to the high frequency of nucleotide insertions or deletions in defined genes, resulting mutated peptides may be also used as shared public neoantigens possibly of use for a broader patient population (34). Of note, the fraction of human leukocyte antigen (HLA) class I bound peptides derived from non-canonical reading frames was found to comprise 10% of all ligands identified on the surface of an expanded B-cell line (35) and thereby provides an additional highly interesting source of TSA as recently summarized (36).

Chromosomal translocations may lead to expression of novel epitopes spanning the respective breakpoint mutation, therefore, representing another source of potential neoantigens. Analyses of immune responses against such neoantigens have provided encouraging rational for clinical applications (37, 38). However, in case of the Philadelphia chromosome defined t(9;21) bcr/abl translocation, vaccination studies have shown variable efficacy (39, 40). One reason might rely in limitations of natural processing of the expected mutated ligands (41). Thus, further studies are required to investigate this anticipated group of highly attractive neoantigens.

Besides the above described sources of altered peptides, B-cell derived malignancies inherit an exceptional source of potentially immunogenic tumor-specific peptides spanning the monoclonal hypervariable recombined immunoglobulin-coding region (42). It has been recently shown for lymphoma that such idiotype-derived ligands are actually presented by MHC class II molecules as detected by mass spectrometry (MS)-based immunopeptidomics and that these are immunogenic (43).

Tumor-specific antigenic peptides may additionally derive from cellular processes specifically altered in tumor cells resulting in a modified peptide repertoire presented by MHC complexes on the tumor surface. Examples comprise peptides with posttranslational modifications as phosphorylation and deamidation potentially resulting in TSA (44–46). Moreover, tumor-specific peptides may derive from alternative splicing in the proteasome (44, 47, 48). As it has been recently reported that spliced peptides substantially contribute to the immunopeptidome (49), it might be highly attractive to more comprehensively investigate the cancer-related MHC peptide ligandome for the presence and immunogenicity of such peptides. However, peptide ligands derived from altered cellular processes currently require MS for detection and there are no algorithms for reliable prediction of such antigens. Moreover, it will be important to investigate in larger studies if these peptides represent really unique TSA suitable for therapeutic targeting approaches.

## Identification of Tumor-Specific Neoantigens

Neoantigens have been primarily identified on the base of defined T-cell responses resulting in a qualitative view on relevant antigens (10, 11). However, general rules could not be deduced from these early reports. Large-scale analyses of genomes and immunopeptidomes, advanced computational analyses, and development of bioinformatics algorithms to predict immunogenicity of tumor-specific peptide ligands greatly enhanced the field (50, 51). This approach resulted in the successful identification of neoantigens in a diversity of malignant diseases although the number of positive hits validated by respective T-cell responses was highly diverse (15, 52–55). Differences of tumor entities as well as inter- and intraindividual heterogeneity of tumors, metastases, and interrogated T-cell repertoires may play an important role for the diversity in the validation rate of predicted epitopes. However, additional aspects govern the quality of such predictions. Technical features as the depth of sequencing and the quality of tumor material, source material for sequencing and algorithms used for SNV calling may have a major impact on the results (56–58). In addition, prediction algorithms for more frequent HLA alleles provide superior results in comparison to less frequent HLA alleles emphasizing the need of larger training datasets (59). Besides, different pipelines for HLA binding prediction have been developed and are currently used in parallel leading to limited comparability of obtained results (60). Moreover, reliable prediction algorithms are currently missing for many aspects of antigen processing and presentation apart from peptide binding. However, there are approaches to improve and harmonize current epitope predictions. A recent implementation of several steps of analysis into one single tool called MuPeXI was provided aiming at integration of predictions and data processing into one straightforward pipeline (61). Application of newly gained knowledge derived from large-scale analyses of pre-existing datasets, such as the pan-cancer analysis of tumor-specific alterations caused by insertions and deletions (33) will further improve our understanding of tumor-specific changes on the genomic level, thereby steadily broadening the current view of potential immunogenic features. Moreover, the bias of epitope prediction may be circumvented by therapeutic approaches as vaccinations based on long peptides or RNA fragments encompassing several point mutations. Two such approaches used in early clinical trials have recently shown encouraging results (20, 21).

Direct identification of mutated peptide ligands by immunoprecipitation of peptide-HLA-complexes and subsequent peptide ligand analysis by MS provides a promising tool for a more straightforward approach with the perspective to define especially those neoepitopes that are indeed well presented on the tumor cell. Feasibility of the detection of naturally presented mutated HLA ligands by this technology has been primarily shown for murine tumors (52, 62) and human cell lines (63). Improved sensitivity as well as optimized bioinformatics algorithms resulted also in the identification of neoantigens directly eluted from primary human tissues (43, 59). In addition, MS data may help to improve current prediction algorithms (64, 65). Feeding of databases such as IEDB and the human immunopeptidome project of the human proteome organization (66) with experimental data is, therefore, of fundamental importance. However, technical issues as requirement for large amounts of tumor material, low yield in peptides after immunoaffinity purification, limited reproducibility and biases from fragmentation methods currently represent major limitations (66). Improvements in this field will likely have a great impact on neoantigen identification to be used for personalized therapies.

## Validation of T-Cell Responses Against Neoantigens

As described above, the identification of all putative mutations within the entire exome (67, 68) paved the way to systematic screens of T cells for respective responses. Pushing the development of technologies for rapid assessment of neoantigen-specific T-cell responses, groundbreaking studies mainly focused on diseases with high mutational burden, especially melanoma and non-small cell lung cancers (50, 54, 69, 70). However, some malignancies with comparably low amounts of tumor-specific mutations also elicit mutation-specific immune responses, including cervical, gastric, and triple-negative breast cancers (55, 71, 72).

As a fairly straightforward approach, the exact expected epitope or longer peptides to be processed by dendritic cells (DCs) are synthesized and screened for recognition by tumor-specific T cells (73). As another possible strategy, T-cell populations may be identified using MHC multimers containing the expected epitope of respective mutated antigens (70, 74, 75). However, MHC multimer analyses may have limitations for fine characterization of neoantigen-specific T-cell populations and may differ to in-depth functional T-cell analyses (59). As an alternative to long peptides, which have to be processed by professional antigen-presenting cells, minigenes comprising respective mutation can be transduced and used for large-scale screening approaches, again circumventing the need of knowing the exact epitope (50). Still, the exact epitope has yet to be determined in additional screenings in case that further characterization of specific immune responses is desired (72, 73). Patient-derived tumor cell lines or spheroids can be used for screening of neoantigen-specific reactivity, although stable expansion of *in vitro* cultures starting with primary human material is often not successful.

The therapeutic potential of targeting somatic mutations throughout vaccination approaches has been also investigated *in vivo* using different mouse models. Specific immune responses could be elicited and successful tumor shrinkage has been observed after application of neoantigen vaccines (67, 68, 76). However, results obtained with murine models rather serve as a proof of principle for a defined immunotherapeutic approach. Another possibility for screening of personalized neoantigen-specific T-cell responses may be achieved by the establishment of individual patient-derived xenografts (PDX). It has been shown that the clonal architecture of patient tumors transplanted in murine hosts exhibit a clonal architecture comparable to tumors grown in the patient (77–80). Therefore, PDX mirror escape mechanisms, which may be translated into the clinical setting. However, some limitations within this approach including changes in the tumor microenvironment and the long time it takes to grow individual xenografts (81) currently prevent larger applications of PDX models in prompt and patient-resembling immunogenicity assessments.

## Sources of Neoantigen-Specific T Cells

For the above described validation of altered target structures, different TCR repertoires may be interrogated. The application of checkpoint inhibitors unleashing the patient’s own immune system emphasized the inherited potential of autologous immune cells to fight cancer. Numerous studies have confirmed neoantigen-specific reactivity within the TIL repertoire (51, 53, 55, 59, 73, 82). In addition, immune responses against mutated peptide ligands can be also detected in the peripheral blood of cancer patients (59, 83) and responses overlapping between PBMC-derived lymphocytes and TIL have been additionally reported (54). Investigation of the TCR beta repertoire of tumor patients vaccinated with a DC vaccine after treatment with Ipilimumab suggested a promotion of neoantigen-specific diversity in TCR beta usage and clonal composition (18). As another important aspect, analysis of treatment-naïve patients in comparison to patients with previous immunotherapies is expected to help to decipher clinically relevant immunoreactivity (84, 85).

For those patients lacking endogenous tumor-specific immune responses or harboring terminally exhausted T cells, the investigation of alternative TCR repertoires provides a meaningful source to empower the patient’s immune system (86). Neoantigen-specific T cells can be also isolated from HLA-matched healthy donors (59, 87). The xenogeneic source of murine TCR (e.g., isolated from HLA-transgenic mice) may provide an alternative source for neoantigen-specific TCR (88). As such, a xenogeneic model is generally rather easily accessible, it may be used to build up a robust workflow for patients lacking specific immune responses. However, it remains questionable, whether this approach confers a significant advantage for neoantigens, as HLA-matched healthy donors should inherit comparable high chances for detectable antigen-specific T-cell frequencies due to circumvention of thymic depletion. Moreover, there might be an enhanced risk for toxicity due to crossreactivity against human peptide ligands, which are not processed or presented by the murine immunopeptidome. However, both repertoires may serve as base for genetic engineering of neoantigen-specific TCR to be used for the adoptive transfer of redirected T cells. Further improvements regarding cost efficacy and time restrictions might enable an automated production of redirected neoantigen-specific T-cells.

Not only the mere detectability of neoantigen-specific T cells, but also the quality of respective T-cell responses is currently under detailed investigation. Various aspects, such as the frequency, phenotype, functional capacities, dynamic changes during clinical course, and the contribution of CD4^+^ and CD8^+^ lymphocytes to tumor rejection (21, 75, 89), are taken into consideration. These analyses may help to understand qualitative characteristics of neoantigens representing immunodominant and suitable rejection antigens inducing an effective T-cell mediated tumor reactivity.

## Future Challenges and Clinical Implications

With respect to neoantigen-targeted therapies but also biomarker development, one central question relies in the selection of those neoantigens, which are in fact relevant in the clinical setting. In this regard, the presence of clonal versus subclonal neoantigens may be highly relevant and tumor heterogeneity may represent a major hurdle for an effective anti-tumor response (70, 90, 91). Driver mutations clearly represent a highly attractive group of potential neoantigens to be targeted for neoantigen-specific therapies as targeting such antigens may limit or decelerate immune evasion due to their frequent clonal nature (17, 19, 92). However, other alterations as genetic changes of tumor cells affecting antigen processing and presentation may still result in immune evasion (19). In fact, defects in antigen presentation incorporate a major risk for immune escape and represent a frequent form of acquired resistance in a diversity of immunotherapies (93–96). A multivariate analysis support the notion of multiple determinants being responsible for the therapeutic outcome (97). A recent study by Riaz and colleagues investigates changes in the tumor evolution and the tumor microenvironment under immune checkpoint inhibition and thereby emphasizes the interdependence of the tumor mutanome and TIL composition (85). In this regard, the assessment of primary and secondary resistance to immune-mediated therapies may potentially lead to improved identification of those patients who may primarily profit from immunotherapies alone and those who may need additional therapeutic approaches. Strategies to restore antigen presentation to be used in combinatorial treatment approaches may become particularly important including the sequential or consecutive application of innovative and well-established therapies as recently reviewed (96, 98). A systematic approach of TCR repertoire profiling across different tumor regions in lung adenocarcinoma hints toward a complex interaction between intratumoral heterogeneity and distribution patterns of clonal T cells (99). In combination with further functional dissection of tumor-specific TCR, information of spatial distribution of neoantigen-specific T cells will likely provide important insights into the dynamics and interactions of tumors and respective neoantigen-specific T-cell responses.

Future directions may, therefore, aim at the comprehensive analysis of immunogenic potential of respective neoantigens by interrogation of diverse repertoires and building up multi-omics and large screening libraries. Therefore, combinatorial analyses of tumor-derived mutations and other molecular characteristics of the tumor cells, tumor microenvironment, and respective immune responses are required for a better understanding of tumor dynamics and selection of suitable structures capable to induce tumor rejection.

## Author Contributions

EB and AK wrote and critically revised the manuscript.

## Conflict of Interest Statement

The authors declare that the research was conducted in the absence of any commercial or financial relationships that could be construed as a potential conflict of interest.
